# Anti-nuclear matrix protein 2 antibody-associated juvenile dermatomyositis with gastrointestinal perforations was successfully treated with traditional therapeutic drugs combined with vedolizumab: a case report after a long-term follow-up and a review of the literature

**DOI:** 10.3389/fped.2024.1522559

**Published:** 2025-01-20

**Authors:** Xue-mei Xu, Shuang Ye, Ying-ying Jin, Chen-xi Liu, Sheng-fang Bao, Hua Huang, Fei Ding, Zhen Yang, Yan-liang Jin

**Affiliations:** ^1^ Department of Rheumatology and Immunology, Shanghai Children’s Medical Center, School of Medicine, Shanghai Jiao Tong University, Shanghai, China; ^2^ Department of Rheumatology, Shanghai Jiao Tong University School of Medicine Affiliated Renji Hospital, Shanghai, China

**Keywords:** juvenile dermatomyositis, gastrointestinal perforation, gastrointestinal involvement, abdominal pain, gastrointestinal bleeding, vedolizumab

## Abstract

Gastrointestinal perforation in patients with juvenile dermatomyositis has been reported as a life-threatening complication in the literature. However, effective treatment of juvenile dermatomyositis with gastrointestinal perforation remains challenging. We report the case of a patient who developed intestinal perforation 5 months after being diagnosed with anti-nuclear matrix protein 2 antibody-positive juvenile dermatomyositis. We systematically reviewed the literature on the medical and/or surgical treatment of gastrointestinal perforation in juvenile dermatomyositis. In addition to our case, as of October 2023, we identified 29 cases of gastrointestinal perforation in patients with juvenile dermatomyositis. Current treatment options for gastrointestinal perforation in juvenile dermatomyositis mainly include corticosteroids, methylprednisolone pulses, rituximab, intravenous immunoglobulin, cyclophosphamide, methotrexate, cyclosporine A, mycophenolate mofetil, and other traditional disease-modifying anti-rheumatic drugs. Notably, juvenile dermatomyositis complicated by gastrointestinal perforation is always associated with disease severity and activity. However, these are extremely severe patients who may not respond to treatment with methylprednisolone pulses and rituximab. Given the limited efficacy of conventional high-intensity systemic immunosuppressive therapy for juvenile dermatomyositis with gastrointestinal perforation, there is an urgent need to explore novel therapeutic approaches. We report on the successful use of vedolizumab in combination with corticosteroids, cyclophosphamide, and intravenous immunoglobulin as a novel therapeutic strategy for treating juvenile dermatomyositis complicated by gastrointestinal perforation. Importantly, up to now, there has been no report of juvenile dermatomyositis with gastrointestinal perforation treated with vedolizumab combined with traditional disease-modifying anti-rheumatic drugs in children.

## Introduction

Juvenile dermatomyositis (JDM) is a rare inflammatory myopathy affecting not only muscles and skin but also other organs, including organs of the cardiovascular, respiratory, and gastrointestinal (GI) systems. GI perforation in patients with JDM has been reported in the literature as a life-threatening complication, causing considerable mortality (1, 2). It has been reported that the anti-nuclear matrix protein 2 (anti-NXP2) antibody may be one of the most common myositis-specific autoantibodies (MSAs) in JDM, which has been reported to be associated with GI involvement (3). Treatment approaches for GI perforations mainly include corticosteroids (CS), methylprednisolone pulses, rituximab (RTX), intravenous immunoglobulin (IVIG), cyclophosphamide (CyC), methotrexate (MTX), cyclosporine A (CSA), mycophenolate mofetil (MMF), and other traditional disease-modifying anti-rheumatic drugs (DMARDs). At present, there is no effective treatment strategy for JDM-associated GI perforation.

## Case report

**Figure 1:**
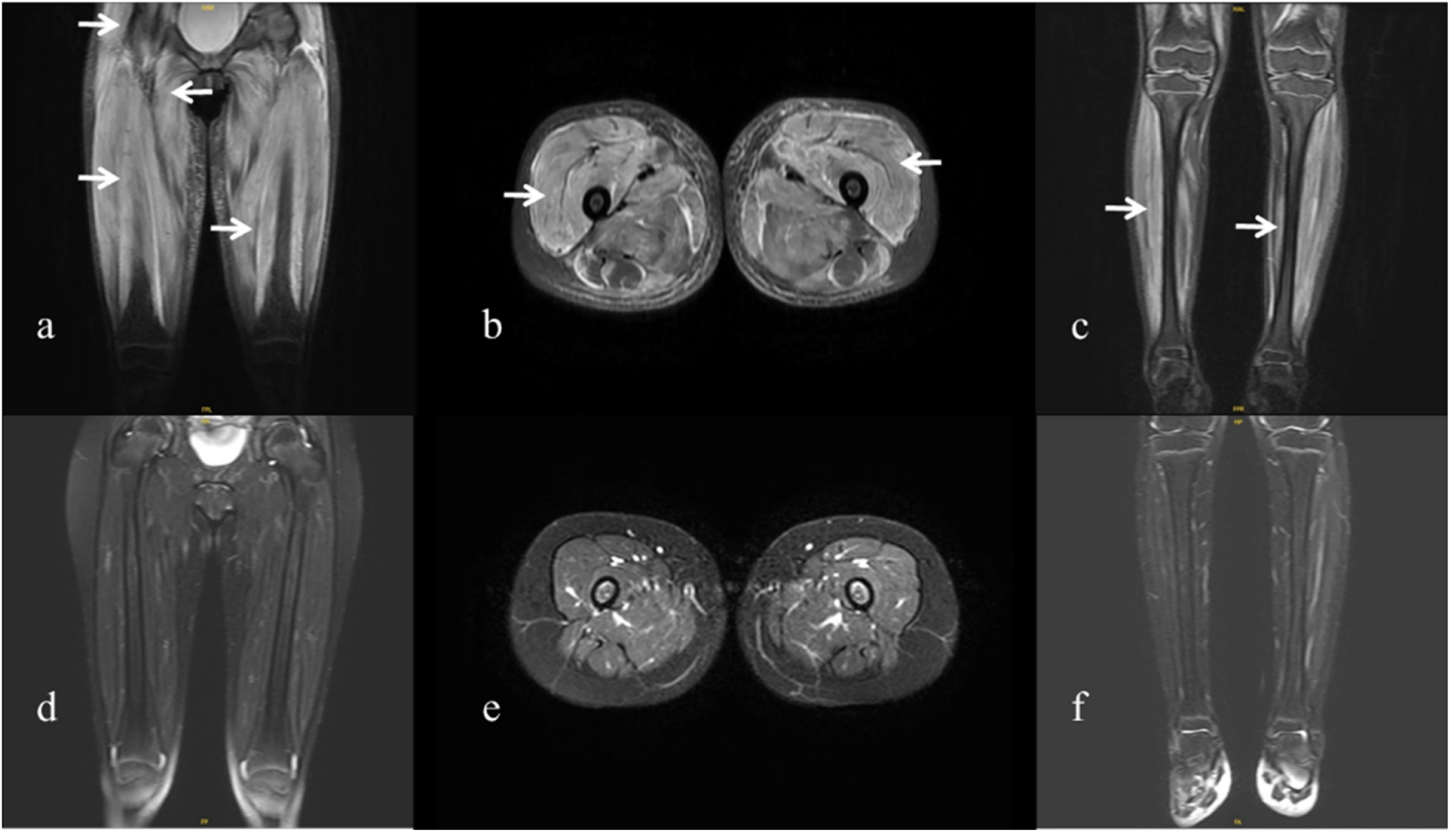
Magnetic resonance imaging of the patient. (**a–c**) Magnetic resonance imaging of the extremities was performed at the onset of the disease, showing diffuse muscle involvement. Coronal and axial T2-weighted fat-saturated images of the pelvis and thigh show bilateral symmetrical involvement of the gluteal, pelvic girdle, and thigh muscles (arrows). (**d–f**) Magnetic resonance imaging performed 1 year after the onset of the disease shows improvement in the patient’s extremities.

**Figure 2:**
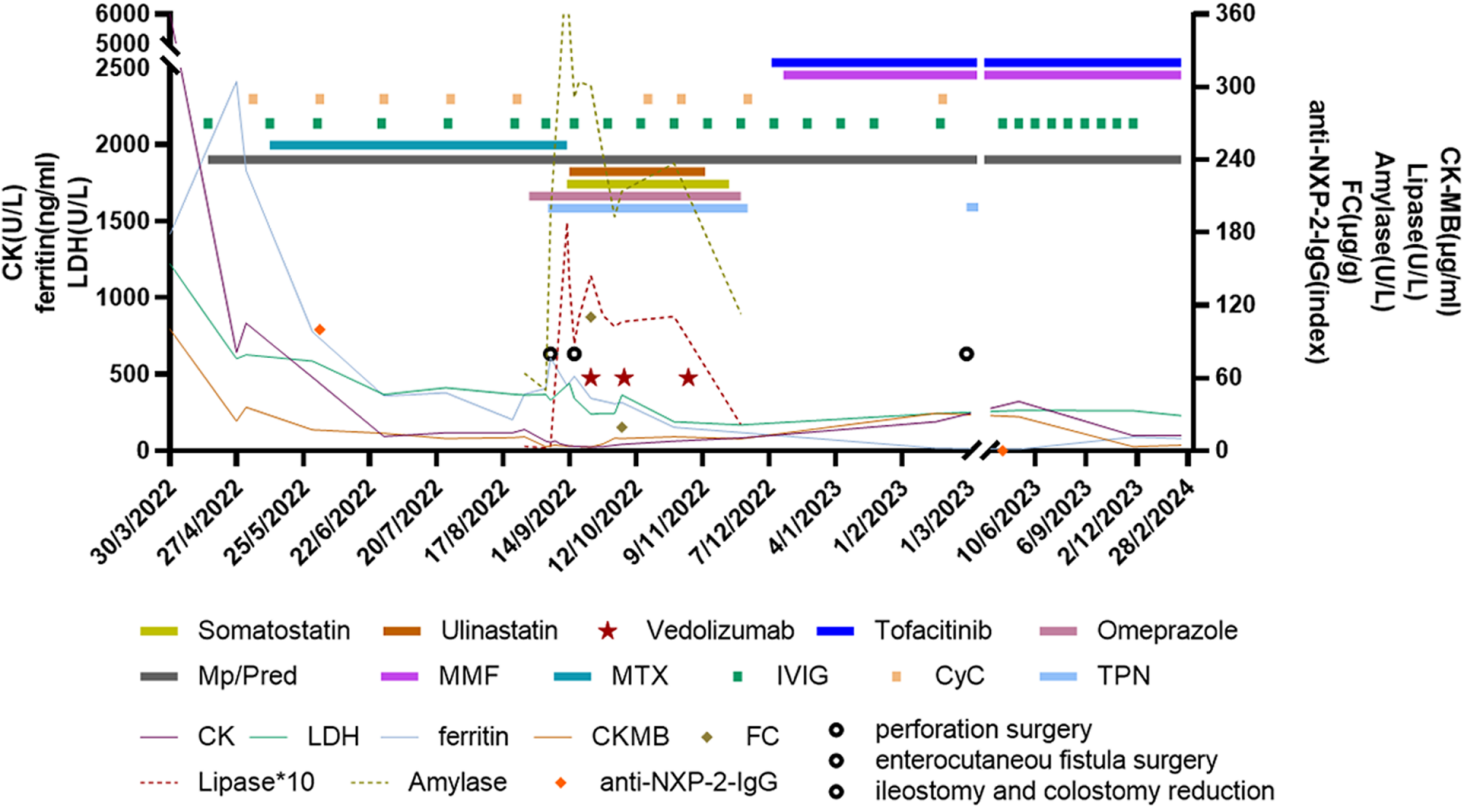
Clinical course and treatment of the patient. Vedolizumab treatment was started at a dosage of 6 mg/kg/dose at 1, 3, and 7 weeks after reoperation. Mp, methylprednisolone; Pred, prednisolone; MMF, mycophenolate mofetil; MTX, methotrexate; IVIG, intravenous immunoglobulin; CyC, cyclophosphamide; PN, parenteral nutrition; CK, creatinine kinase; LDH, lactate dehydrogenase; CK-MB, creatine kinase isoenzyme MB; FC, fecal calprotectin; NXP2, anti-nuclear matrix protein 2.

A previously healthy 6.5-year-old girl presented with muscle weakness and intermittent fever for more than 1 month before being admitted to the hospital on 30 March 2022. She presented with fever, muscle weakness, palmar erythema, and a heliotrope rash over her eyelids. Laboratory results revealed the following findings: anti-NXP2 antibody: 100 (normal: 0–5) index, creatine kinase (CK): 6:031 (normal: 30–135) U/L, lactate dehydrogenase (LDH): 1:221 (normal: 120–246) U/L, aspartate aminotransferase (AST): 321 (normal: 15–46) U/L, alanine aminotransferase (ALT): 100 (normal: <35) U/L, CK isoenzyme MB (CK-MB): 100.3 (normal: <3.7) μg/L, myohemoglobin (Mb): 889.6 (normal: 11.6–73.0) ng/ml, erythrocyte sedimentation rate (ESR): 59 (normal: 0–20) mm/h, ferritin: 1:415.2 (normal: 4.6–204.0) ng/ml, and interleukin-6: 16.86 (normal: 0–5.4) pg/ml. Magnetic resonance imaging of the patient’s extremities suggested myositis (Figures 1a–c). She was diagnosed with definite idiopathic inflammatory myopathies (JDM subgroup) based on the 2017 European League Against Rheumatism/American College of Rheumatology (EULAR/ACR) classification criteria for adult and juvenile idiopathic inflammatory myopathies. Her Childhood Myositis Assessment Scale (CMAS) score was 14/52. The patient received combination therapy including prednisone (2 mg/kg/day, gradually tapered), five courses of CyC (500 mg/m^2^/dose at 4-week intervals), IVIG [2 g/kg/dose × 3, 1 g/kg/dose × 3], oral MTX (7.5 mg weekly), and hydroxychloroquine (HCQ; 100 mg daily) for nearly 5 months (Figure 2). She also received trimethoprim/sulfamethoxazole as prophylaxis for opportunistic infections. LDH reduced to 371 U/L, CK-MB reduced to 10.9 µg/L, ALT reduced to 59 U/L, AST reduced to 57 U/L, and CK, Mb, ESR, and ferritin returned to normal. There was no significant improvement in muscle strength and CMAS score. However, intermittent abdominal pain and fever developed 5 months after the onset of the disease.

**Figure 3:**
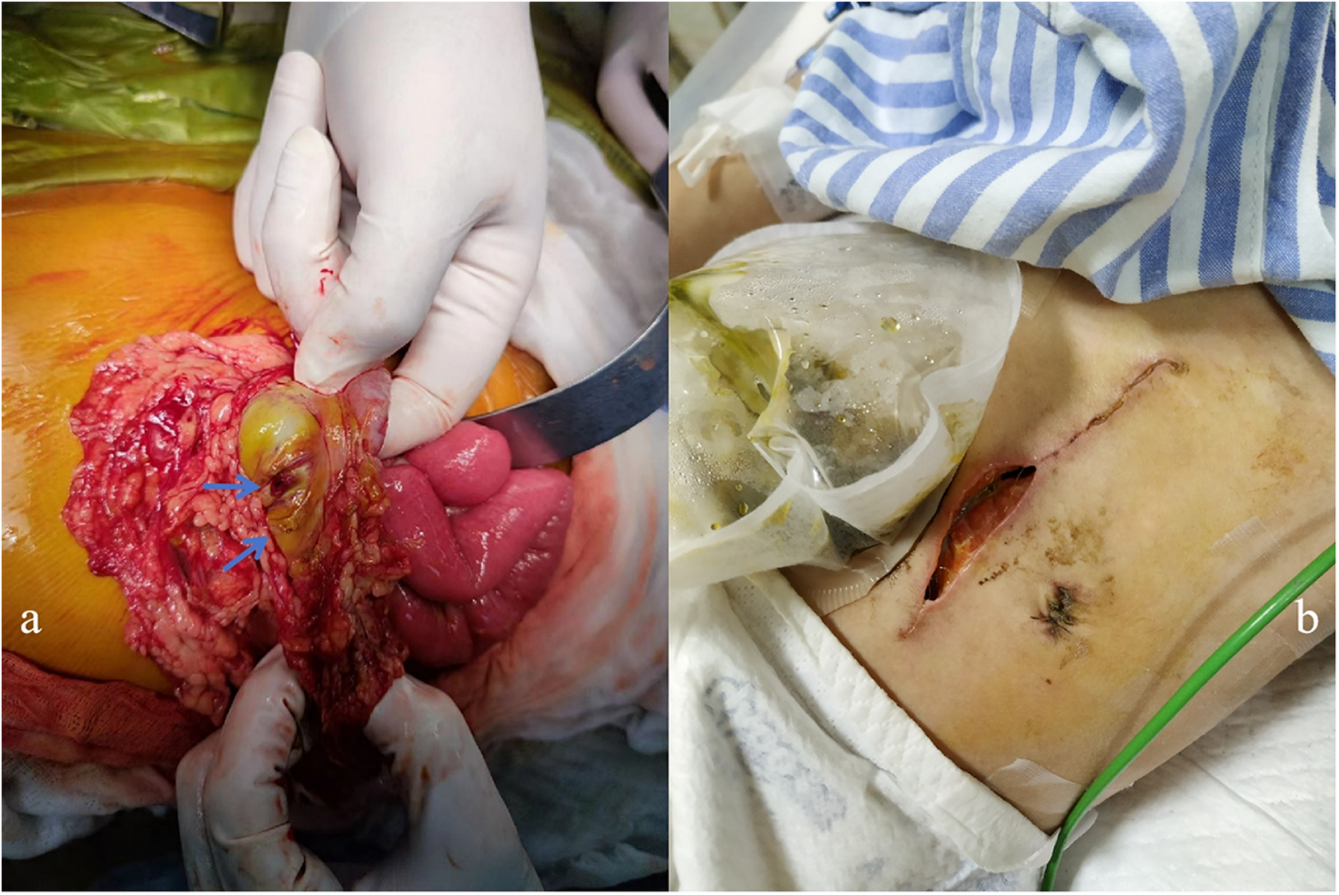
(**a**) Two 5-mm perforations of the transverse colon were observed during surgery. (**b**) The patient developed an enterocutaneous fistula 10 days after surgery.

The patient had abdominal pain for 3 days and a low-grade fever (37.9℃) once before being readmitted on 26 August 2022. Her medications prior to admission were prednisone 25 mg daily, MTX 7.5 mg weekly, folic acid 5 mg weekly, HCQ 100 mg daily, CyC 500 mg/m^2^/dose at a 4-week interval, and IVIG 1 g/kg monthly. At first, the patient had a *Clostridium difficile* infection diagnosed using a stool polymerase chain reaction test. As her condition was not improving after antibiotic therapy, an erect abdominal x-ray and computed tomography (CT) were conducted, which showed no abnormalities. Endoscopy and colonoscopy showed no abnormal findings. The patient presented with recurrent vomiting, fever, and abdominal pain. On 6 September 2022, the patient developed tachycardia in the absence of a fever and worsening abdominal pain. Physical examination showed severe proximal muscle weakness, and an abdominal palpation was refused. Blood parameters revealed that procalcitonin (1.93 ng/ml), C-reactive protein (148 mg/L), and serum amyloid A (245.7 mg/L) increased significantly and that white blood cell count decreased (2.77 × 10^9^/L). Fecal calprotectin [110.3 (normal: 0–50.0)] was also elevated. The erect abdominal X-ray revealed gas under the diaphragm. During surgery, two 5-mm perforations in the transverse colon were observed (Figure 3a); the perforations were repaired, and an ileostomy was performed. Histopathology of the resected bowel wall showed no signs of vasculitis. The patient gradually resumed eating after the operation in the pediatric intensive care unit (PICU). However, 1 week later, she developed further severe abdominal pain. Due to elevated amylase and lipase, pancreatitis was suspected. On postoperative day 10, she developed an enterocutaneous fistula (Figure 3b). Reoperation showed necrosis and erosion of the bowel at the perforation of the transverse colon. Approximately 7 cm of the transverse colon was removed, and a colostomy and ileostomy were performed.

Considering the severe GI involvement, vedolizumab (VDZ; 6 mg/kg/dose 1, 3, and 7 weeks after reoperation) was added to the patient's medication regimen. Other treatments included total parenteral nutrition, intravenous methylprednisolone (1 mg/kg/day), IVIG (1 g/kg/dose at 2-week intervals), five courses of CyC (250 mg/m^2^/dose × 2, 500 mg/m^2^ × 3) followed by MMF, and fresh frozen plasma infusion to manage intestinal edema. MTX was discontinued during this period. Parenteral nutrition was gradually restored 1.5 months later. She regained the ability to walk and dance 1 and 2 months, respectively, after surgery. Tofacitinib (Tofa) was introduced 3 months post-surgery, and prednisone, MMF, and IVIG were maintained. The patient underwent ileostomy reduction and colostomy reduction 5 months after surgery (28 February 2023). She recovered well and experienced no complications. Prednisone was reduced to 5 mg/day 13 months after surgery. Magnetic resonance imaging revealed remarkable improvement in her extremities (Figures 1d–f), and the patient tested negative for anti-NXP2 antibody 1 year after disease onset. Her disease is now managed with prednisone 5 mg every other day, Tofa 10 mg/day, and MMF 500 mg/day; IVIG was discontinued. A physical examination revealed normal muscle strength and no recurring rash. Her CMAS score was 49/52.

## Methods

A systematic search was performed to retrieve all original publications, including case reports, case series, case-based reviews, and review articles on treatment options for patients with JDM with GI perforation. PubMed was searched using the terms “juvenile dermatomyositis,” “gastrointestinal perforation,” “gastrointestinal involvement,” and “abdominal pain.” Articles involving patients with juvenile dermatomyositis that resulted in gastrointestinal perforation were included. We searched all English-language articles up to October 2023. In addition to our case, we identified 20 articles with 29 relevant cases describing gastrointestinal perforation in patients with juvenile dermatomyositis (2, 4–22). Relevant articles and their references were checked. Information obtained systematically included demographics, clinical manifestations, medications, surgical management, and outcomes.

## Results

**Figure 4:**
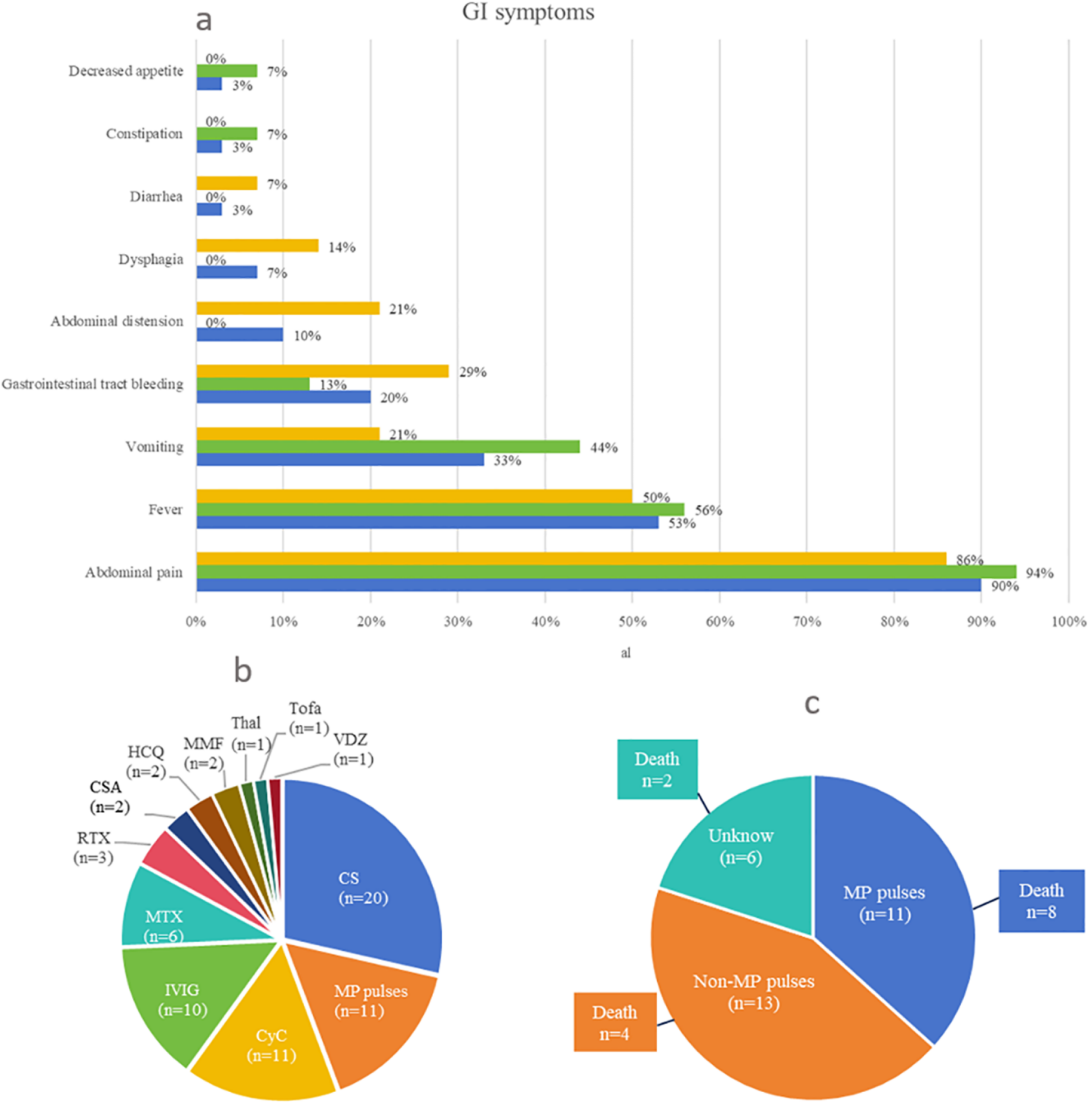
(**a**) Clinical manifestations of the first gastrointestinal perforation. Treatment for juvenile dermatomyositis with gastrointestinal perforation: (**b**) If a patient had been treated with more than one drug, we counted all of them in this graph. (**c**) Relationship between treatment type and death. In this study, 14 patients died. With the exception of 2 patients for whom treatment information was missing, 8 of the 12 patients who died were treated with MP pulses. CS, corticosteroids; MP, methylprednisolone; CyC, cyclophosphamide; IVIG, intravenous immunoglobulin; MTX, methotrexate; RTX, rituximab; CSA, cyclosporine A; HCQ, hydroxychloroquine; MMF, mycophenolate mofetil; Thal, thalidomide; Tofa, tofacitinib; VDZ, vedolizumab.

Overall, 10 of the 30 patients (including our case) were boys and 18 were girls. Gender was not mentioned in the two cases. The age of onset of JDM and first GI perforation for the patients ranged from 3.25 to 13 years (mean ± SD: 7.81 ± 2.93 years) and 3.75 to 20 years (mean ± SD: 9.03 ± 3.79 years), respectively. The period from JDM onset to GI perforation for the patients ranged from 0 to 108 months [7.50 (3.75–13.50) months]. The age of onset of JDM and first GI perforation in female patients ranged from 3.25 to 9.75 years (mean ± SD: 7.09 ± 2.74 years) and 3.75 to 9.79 years (mean ± SD: 7.96 ± 2.88 years), respectively. The period from JDM onset to GI perforation in female patients ranged from 0 to 48 months [6.00 (3.75–12.00) months]. The age of onset of JDM and first GI perforation in male patients ranged from 4 to 13 years (mean ± SD: 8.55 ± 3.17 years) and 4.17 to 15.83 years (mean ± SD: 10.51 ± 4.99 years), respectively. The period from JDM onset to GI perforation in male patients ranged from 0 to 108 months [11.00 (2.38–30.00) months], most of which occurred within 1 year. Our collected cases were almost evenly distributed in terms of age between 3 and 12 years. The most common initial symptoms of GI perforation were abdominal pain (26/30, 87%) and fever (16/30, 53%) (Figure 4a). Locations of perforations included duodenum (63%, 19/30), colon (37%, 11/30), esophagus (17%, 5/30), jejunum (10%, 3/30), stomach (7%, 2/30), ileum (3%, 1/30), and cecum (3%, 1/30); perforations at multiple sites or recurrent perforations were reported in 12 patients (40%, 12/30). When GI perforation occurred, activated muscle and skin involvement presented in 63% (19/30) and 67% (20/30), respectively, of patients with NXP2 ^+^ JDM. The antibody panel was not described in most of the cases, especially in earlier cases. Only 8 of 30 patients underwent testing for myositis-specific antibodies, with the anti-NXP2 antibody being positive in 7 patients. Endoscopy was performed in 17% (5/30) of patients, and spontaneous perforation was observed in 40% (2/5) of these patients. Imaging findings during GI perforation included the following: nine patients had abnormal X-ray results, seven patients had abnormal CT results, one patient had abnormal ultrasound results, five patients had no imaging abnormalities, and information on imaging was not available in seven patients. Repeated x-ray, CT, or ultrasound examination revealed GI perforation in nine patients. Figure 4b shows the treatment for JDM with GI perforation in 24 patients, excluding 6 patients with no treatment information. Glucocorticoids were used in 20 patients, 11 of whom received methylprednisolone pulse treatment. Furthermore, 11 patients were treated with CyC, 10 patients were treated with IVIG, 6 patients were treated with MTX, 3 patients were treated with RTX, 2 patients were treated with CSA, 2 patients were treated with HCQ, 2 patients were treated with MMF, 1 patient was treated with thalidomide, 1 patient was treated with Tofa, and 1 patient (our patient) was treated with VDZ. The mortality rate of JDM with GI perforation was 47% (14/30). With the exception of 2 patients for whom treatment information was missing, 8 of the 12 patients who died were treated with methylprednisolone pulses (Figure 4c). Surgical measures were undertaken in 96% (24/25) of patients; surgical repair could not be undertaken in one patient due to unstable vital signs, severe infection, and severe and extensive intestinal lesions. Surgical management information was not provided for five patients.

## Discussion

GI perforation in patients with JDM has been reported as a life-threatening complication in the literature. In this study, we collected information on 30 patients with JDM who experienced GI perforation (24–22). The identification of MSAs has facilitated the characterization of individual subgroups of children with specific phenotypes of JDM (23). Notably, the presence of anti-NXP2 antibodies has been associated with more severe forms of JDM, particularly those involving GI manifestations, as previously described (2, 24). In our center, there were a total of 12 patients with NXP2 ^+^ JDM, of whom 42% (5/12) presented with GI involvement, and one patient developed intestinal perforation. However, previous literature did not mention the detection of MSAs; recent studies have shown that seven out of eight patients with JDM complicated by GI perforation tested positive for anti-NXP2 antibodies (19, 21, 22), as described in other reports (25). Our study showed that the mortality rate of JDM with GI perforation was 47%, which is surprisingly consistent with that reported in adults (25). Therefore, it is crucial to remain vigilant for severe GI complications in NXP2 ^+^ JDM during disease progression. A total of 77% of GI perforations occurred within 1 year of JDM diagnosis. The most common initial symptoms were abdominal pain and fever, along with disease activity and severity. Meanwhile, the differential diagnosis of fever and abdominal pain should be considered.

In our study cohort, 50% (9/18) of patients with imaging abnormalities underwent repeat evaluation. Based on our experience, repeated, frequent examinations by the pediatrician and surgeon with a comparison of findings are very helpful in the decision-making process and are necessary to confirm or exclude this diagnosis. The upper and lower GI tract can be affected. The duodenum was the most common site of perforation (2). Endoscopic evaluation is rarely utilized in clinical practice. In our reported case, the patient underwent an endoscopy prior to the occurrence of intestinal perforation, but no ulcers or perforations were observed. However, 4 days later, x-rays confirmed the presence of intestinal perforation. Therefore, we speculate that the perforation may have originated from both the muscular and serosal layers before extending to the mucosal layer. The histopathology of the resected bowel wall showed no signs of vasculitis. Nevertheless, it should be noted that vasculitis can manifest focally, and colon perforation can occur due to ischemic changes distant from areas with vascular impairment. The exact etiology of intestinal perforation in JDM remains uncertain.

There is a serious challenge in treating GI perforation. Treatment approaches for GI perforation mainly include CS, methylprednisolone pulses, RTX, IVIG, CyC, MTX, CSA, MMF, and other traditional DMARDs. In the literature, three patients received RTX and only one survived (13, 19, 22), while four patients underwent plasma exchange and only one survived (11, 13, 16, 22). We observed eight deaths in 11 patients treated with methylprednisolone pulses, compared with four deaths in 13 patients treated without methylprednisolone pulses. The number of cases was limited; hence, there was no clear statistical difference (8, 9, 16, 21, 22). These patients may not respond to treatment with methylprednisolone pulses and rituximab, although they are often used for severe/refractory JDM. We hope that a new treatment strategy can be developed to benefit more pediatric patients in the future. In line with this, we have reported the world’s first JDM case with gastrointestinal perforation that was successfully treated with traditional therapeutic drugs combined with VDZ; this study aimed to provide clinicians with an additional treatment option and to verify its effectiveness. VDZ downregulates intestinal inflammation by specifically inhibiting intestinal T-lymphocyte migration into the tissue (25). Therefore, its mechanism of action is restricted to the gastrointestinal tract, which potentially reduces the risk of systemic immunosuppression that leads to increased infection rates. In an attempt to strike a balance between GI immune-mediated processes and vulnerability to infection, VDZ was introduced to block the migration of lymphocytes into inflamed intestinal tissue. It should be emphasized that VDZ has no effect on muscle weakness in patients who use this drug. VDZ treatment was administered 1, 3, and 7 weeks after the intestinal perforation, along with a moderate dose of CS, a low dose of CyC, and IVIG. The patient experienced significant improvement in muscle weakness and regained the ability to walk and dance 1 and 2 months after surgery, respectively. MMF was used as a maintenance therapy for the intestinal perforation, while Tofa was introduced at 3 months post-surgery. Prednisone dosage was gradually reduced to 5 mg every other day with discontinuation of IVIG. In adults, four patients who received this therapy survived after the GI events (26). Combining VDZ with CS, CyC, and IVIG appears to be an effective novel therapeutic approach for managing JDM complicated by intestinal perforations.

## Conclusions

GI perforation is a fatal complication of JDM. It is crucial to take abdominal pain seriously in children with JDM, especially in those with NXP2 ^+^ JDM. VDZ combined with CS, CyC, and IVIG may be a novel effective treatment option for acute JDM complicated by GI perforation, while Tofa may be used for maintenance therapy.

## Data Availability

The original contributions presented in the study are included in the article/Supplementary Material, further inquiries can be directed to the corresponding author.
